# A Machine Learning Approach Using FDG PET-Based Radiomics for Prediction of Tumor Mutational Burden and Prognosis in Stage IV Colorectal Cancer

**DOI:** 10.3390/cancers15153841

**Published:** 2023-07-28

**Authors:** Hyunjong Lee, Seung Hwan Moon, Jung Yong Hong, Jeeyun Lee, Seung Hyup Hyun

**Affiliations:** 1 Department of Nuclear Medicine, Samsung Medical Center, Sungkyunkwan University School of Medicine, 81 Irwon-ro, Gangnam-gu, Seoul 06351, Republic of Korea; nmhjlee@gmail.com (H.L.); 2 Division of Hematology/Oncology, Department of Medicine, Samsung Medical Center, Sungkyunkwan University School of Medicine, 81 Irwon-ro, Gangnam-gu, Seoul 06351, Republic of Korea

**Keywords:** colorectal cancer, FDG PET/CT, radiomics, tumor mutational burden, prognosis

## Abstract

**Simple Summary:**

In this study, we explored the potential of using F-18 fluorodeoxyglucose positron emission tomography (FDG PET), to predict the genetic characteristics and prognosis of patients with stage IV colorectal cancer. We used a machine learning approach to analyze association between image patterns and the tumor mutational burden (TMB), which can provide information for selecting treatment options and predicting prognosis. Our results showed that several radiomic features from the PET images were significantly associated with TMB. Furthermore, we developed a scoring system based on these features, which was found to be a significant predictor of patient survival. This research suggests that FDG PET could be a surrogate marker of TMB in advanced colorectal cancer. It offers a non-invasive method to assess a tumor’s genetic characteristics and predict patient outcomes, potentially leading to more personalized and effective treatment strategies.

**Abstract:**

Introduction: We assessed the performance of F-18 fluorodeoxyglucose positron emission tomography (FDG PET)-based radiomics for the prediction of tumor mutational burden (TMB) and prognosis using a machine learning (ML) approach in patients with stage IV colorectal cancer (CRC). Methods: Ninety-one CRC patients who underwent pretreatment FDG PET/computed tomography (CT) and palliative chemotherapy were retrospectively included. PET-based radiomics were extracted from the primary tumor on PET imaging using the software LIFEx. For feature selection, PET-based radiomics associated with TMB were selected by logistic regression analysis. The performances of seven ML algorithms to predict high TMB were compared by the area under the receiver’s operating characteristic curves (AUCs) and validated by five-fold cross-validation. A PET radiomic score was calculated by averaging the z-score of each radiomic feature. The prognostic power of the PET radiomic score was assessed using Cox proportional hazards regression analysis. Results: Ten significant radiomic features associated with TMB were selected: surface-to-volume ratio, total lesion glycolysis, tumor volume, area, compacity, complexity, entropy, correlation, coarseness, and zone size non-uniformity. The k-nearest neighbors model obtained the good performance for prediction of high TMB (AUC: 0.791, accuracy: 0.814, sensitivity: 0.619, specificity: 0.871). On multivariable Cox regression analysis, the PET radiomic score (Hazard ratio = 4.498, 95% confidential interval = 1.024–19.759; *p* = 0.046) was a significant independent prognostic factor for OS. Conclusions: This study demonstrates that PET-based radiomics are useful image biomarkers for the prediction of TMB status in stage IV CRC. PET radiomic score, which integrates significant radiomic features, has the potential to predict survival in stage IV CRC patients.

## 1. Introduction

Colorectal cancer (CRC) is a representative malignancy of the digestive organs, the third most common malignancy and the second most deadly cancer worldwide [1]. Colectomy is the first treatment option for non-metastatic CRC. Even in metastatic CRC, resection is a recommended option for hepatic or pulmonary metastasis [2]. However, palliative chemotherapy is usually performed in advanced or metastatic CRC. Palliative chemotherapy has shown good cost-effectiveness as well as survival benefit [3,4]. As various chemotherapy agents and immuno-oncologic treatment options have been developed in recent days, the importance of palliative chemotherapy for CRC is increasing [5,6].

F-18 fluorodeoxyglucose positron emission tomography/computed tomography (FDG PET/CT) is a robust imaging modality used to diagnose malignancy and evaluate treatment response of cancer [7,8]. It provides additional information, including presence of regional or distant metastasis for initial staging or evaluation of operability in CRC [9,10]. Furthermore, volumetric parameters such as metabolic tumor volume (MTV) or total lesion glycolysis (TLG) are revealed as excellent prognostic factors of CRC [11,12]. In addition to CT or MR based radiomics [13,14], PET-based radiomics have been highlighted in recent years. Intensity variability, size-zone variability, and entropy from FDG PET/CT showed prognostic potential in patients with surgically resected CRC [15,16]. Previous reports suggested that PET-based radiomics may reflect the heterogeneity of the tumor.

Tumor mutational burden (TMB), the total number of genetic mutations in tumor tissue, is a recently highlighted biomarker for immunotherapy in various malignancies [17]. According to a previous study, high TMB (eight or more gene mutations) has been associated with better recurrence-free survival in CRC patients with surgery followed by adjuvant chemotherapy [18]. In addition, TMB was an independent biomarker to stratify patients for response to the immune checkpoint inhibitor [19]. A previous study investigated the correlation between PET-based radiomics and TMB in lung cancer [20]. Another pan-cancer analysis, including gastrointestinal cancer, showed an association between the standardized uptake value (SUV) on FDG PET/CT and TMB [21]. However, no prior study has attempted to predict TMB status using PET-based radiomics in CRC.

In this study, we aimed to assess the performance of PET-based radiomics for the prediction of TMB using a machine learning (ML) approach in patients with stage IV CRC. In addition, the prognostic significance of a newly developed PET radiomic score was assessed.

## 2. Methods

### 2.1. Subjects and Clinical Data

**Figure 1:**
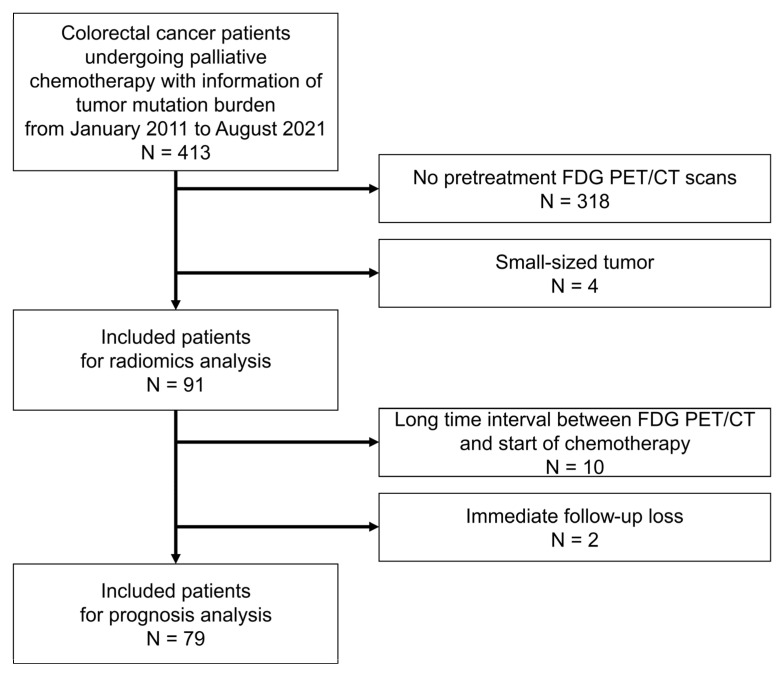
Flowchart of the study design.

Between January 2011 and August 2021, 413 patients who underwent palliative chemotherapy for CRC with information on TMB from pathologic specimens were retrospectively enrolled. Among them, 318 patients without pretreatment FDG PET/CT were excluded. Four additional patients were excluded due to small size tumors less than 64 voxels, which could not produce radiomic feature data in the analysis software. Ultimately, 91 patients were included in this study (Figure 1, 53 males and 38 females; mean age, 58.0 ± 12.2 years).

Clinical information, including sex, age, location of primary tumor, histological type of the primary tumor, chemotherapy, and palliative surgery status, was obtained by reviewing electronic medical records. Palliative chemotherapy was performed when there was a distant metastasis in the initial staging workup. Chemotherapy cycles and duration were same in the group with same regimen unless there was evidence of progressive disease during chemotherapy. Disease progression was determined by oncologists when there was increasing size of malignant lesions or new malignant lesions on serial imaging or pathological studies. The events for overall survival analysis were defined as any cause of death. The survival duration was defined as duration from the start date of palliative chemotherapy to the last follow-up date or death date. Our institutional review board approved this retrospective study (IRB #2022-10-037).

### 2.2. Tumor Mutational Burden

Pre-treatment biopsy specimens were obtained by incisional biopsy (62 cases) or surgical resection (29 cases). TMB was measured by TruSight Oncology 500 (Illumina Inc., San Diego, CA, USA) as described previously [22]. Briefly, 40 ng of DNA was quantified with the Qubit dsDNA HS Assay (Thermo Fisher Scientific, Inc., Waltham, MA, USA) on the Qubit 2.0 Fluorometer (Thermo Fisher Scientific) and then sheared using a Coraris E220 focused-ultrasonicator (Woburn, MA, USA) and an 8 microTUBE–50 Strip AFA Fiber V2 following the manufacturer’s instructions. For DNA library preparation and enrichment, the TruSight Oncology 500 Kit (Illumina) was used following the manufacturer’s instructions. Post-enriched libraries were quantified, pooled, and sequenced on a NextSeq 500 (Illumina). The quality of the NextSeq 500 (Illumina) sequencing runs was assessed with the Illumina Sequencing Analysis Viewer (Illumina). Sequencing data were analyzed with the TruSight Oncology 500 Local App Version 1.3.0.39 (Illumina). TMB was reported as mutations per megabase (Mb) sequenced, and high TMB was defined as more than 10 mutations per Mb (≥10 Mut/Mb).

### 2.3. FDG PET/CT Imaging

All patients fasted for at least six hours and had blood glucose levels of less than 200 mg/dL at the time of their FDG PET/CT scans. Whole-body PET and CT images from the basal skull to mid-thigh were acquired 60 min after the injection of 5.0 MBq/kg FDG without intravenous or oral contrast on a Discovery LS, a Discovery STE, or a Discovery MI DR PET/CT scanner (GE Healthcare, Milwaukee, WI, USA). Continuous spiral CT was performed with an 8-slice helical CT (140 keV, 40–120 mA; Discovery LS) or 16-slice helical CT (140 keV, 30–170 mA; Discovery STE, 120 keV, 30–100 mA; Discovery MI DR). An emission scan was then obtained from head to thigh for 4 min per frame in 2-dimensional mode (Discovery LS), 2.5 min per frame in 3-dimensional mode (Discovery STE), or 2 min per frame in 3-dimensional mode (Discovery MI DR). PET images were reconstructed using CT for attenuation correction by the ordered-subsets expectation maximization algorithm with 28 subsets and 2 iterations (matrix 128 × 128, voxel size 4.3 × 4.3 × 3.9 mm; Discovery LS), 20 subsets and 2 iterations (matrix 128 × 128, voxel size 3.9 × 3.9 × 3.3 mm; Discovery STE), or 18 subsets and 4 iterations (matrix 192 × 192, voxel size 3.9 × 3.9 × 3.3 mm; Discovery MI DR). SUV was calculated by adjusting for the administered FDG dose and patient body weight.

### 2.4. PET Radiomic Features

PET radiomics extraction was performed using LIFEx software v7.1.13, a freeware for radiomic feature calculation [23]. The target primary tumor was identified by an experienced nuclear medicine physician who was unaware of all clinical information except the target tumor site. Tumor margins were delineated using the Nestle adaptive threshold method (parameter *beta* = 0.3) provided by the software (Supplementary Figure S1). Subsequently, tumor margins were verified by an experienced nuclear medicine physician. After tumor segmentation of the target lesion, the software extracted a total of 157 PET-based radiomics. Among them, we selected 92 radiomic features that were provided as a single real number: 25 conventional features based on SUV data, 12 morphological features, 24 gray-level co-occurrence matrix (GLCM) features, 11 gray-level run length matrix (GLRLM) features, 5 neighboring gray tone difference matrix (NGTDM) features, and 15 gray-level size zone matrix (GLSZM) features. A detailed list is described in Supplementary Table S1. ComBat harmonization was conducted using package “neuroCombat” in R software to remove batch effects due to various PET/CT instruments [24].

### 2.5. Machine Learning Approach

As a feature selection method, logistic regression analysis was performed to explore PET-based radiomics, which can discriminate between high- and low-TMB groups. Significant radiomic features were selected as imaging features for the prediction of TMB. A false discovery rates (FDR) less than 0.1 was considered statistically significant. As a surrogate imaging biomarker, we calculated a PET radiomic score by averaging the z-score of each selected imaging feature.

Seven ML algorithms were applied to construct a prediction model: generalized linear model (GLM), linear discriminant analysis (LDA), quadratic discriminant analysis (QDA), k-nearest neighbors (KNN), support vector machine (SVM), neural network (NN), and random forest (RF). A discriminative performance of the ML model was measured by area under the receiver operating characteristic (ROC) curve, accuracy, sensitivity, specificity, F1 score, and precision. To assess model generalizability and select an appropriate model, we used a five-fold cross-validation method. Hyperparameter tuning was performed using grid search on cross-validation by package “caret” in R software. The model with the highest accuracy was selected as the representative model for each ML algorithm.

### 2.6. Statistical Analysis

Pearson’s correlation analyses were performed to reveal associations between PET-based radiomics and TMB. T-tests were performed to explore PET-based radiomics, which demonstrated significant differences between the high- and low-TMB groups.

Prognostic validation was performed in 79 patients; 10 patients with long time intervals more than 60 days between the initial FDG PET/CT scan and start of palliative chemotherapy were excluded, and two patients were excluded due to loss to follow-up (Figure 1).

Clinical variables, including sex, age, location of primary tumor, histological type, regimen of chemotherapy, TMB, and PET radiomic score, were employed for Cox proportional hazards regression analysis. Continuous variables were dichotomized into two groups. Age was dichotomized by median value and TMB by the cutoff value of 10 mutations per megabase (Mut/Mb). The PET radiomic score was dichotomized into two groups according to an optimal cutoff value for overall survival (OS). This was explored by the “surv cutpoint” function of the package “survminer” in R software.

Uni- and multivariable survival analyses for OS were used to evaluate the prognostic power of each variable. Hazard ratios (HRs) and 95% confidence intervals were estimated. Log-rank statistics and Kaplan–Meier survival curves were also obtained. All variables without collinearity were included in multivariable survival analysis. All statistical analyses were performed using R software (v. 4.0.4, R Foundation for Statistical Computing, Vienna, Austria). A *p*-value lower than 0.05 was considered statistically significant. FDRs were calculated to correct *p*-values for multiple tests.

## 3. Results

### 3.1. Patients

The patient characteristics are summarized in Table 1. In 88 patients (96.7%), the histological type was adenocarcinoma. One patient (1.1%) was a small cell carcinoma patient. The remaining two patients (2.2%) were unclassified carcinoma patients. In 44 patients (48.4%), palliative surgery was performed: 13 patients before chemotherapy and 31 patients after chemotherapy. Palliative surgery included low anterior resection (19 cases), hemicolectomy (eight cases), anterior resection (seven cases), intersphincteric resection (four cases), etc. In 21 patients (23.1%), tumors with TMBs greater than 10 Mut/Mb were assigned to the high-TMB group. In the remaining 70 patients (76.9%), tumors with TMBs less than 10 Mut/Mb were assigned to the low-TMB group. The median value of TMB was 5.5. TMB ranged from 10.2 to 80.6 (mean: 22.0) for the high-TMB group and from 0 to 9.4 (mean: 5.7) for the low-TMB group. There were no significant associations between TMB and sex, age, histological type, histological grade, chemotherapy regimen, or palliative surgery status.

### 3.2. PET-Based Radiomics and Tumor Mutation Burden

**Figure 2:**
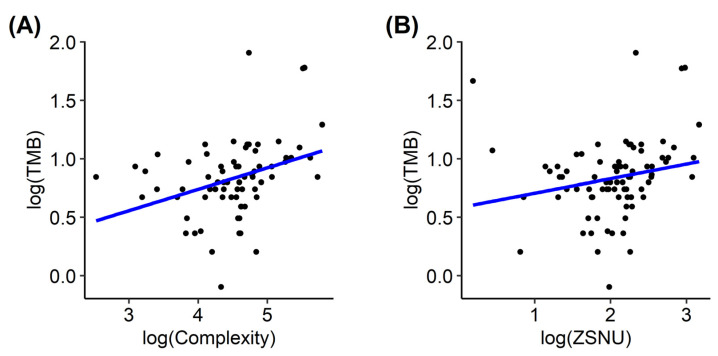
Correlation of PET radiomics with TMB. Complexity ((**A**), *r* = 0.332; FDR = 0.007) and zone size non-uniformity ((**B**), *r* = 0.351; FDR = 0.011) showed significant correlations with TMB. PET, positron emission tomography; TMB, tumor mutational burden; FDR, false discovery rate.

**Figure 3:**
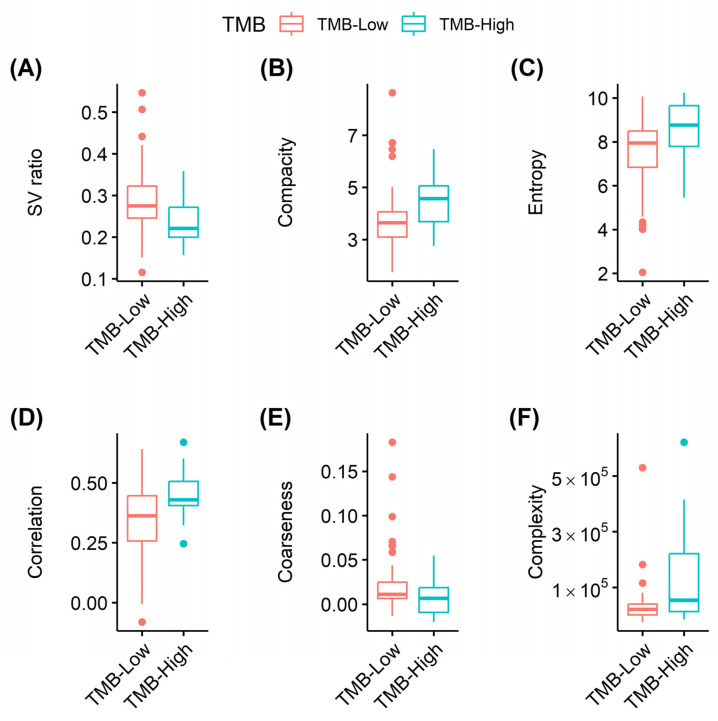
Comparison of PET radiomics between high- and low-TMB groups. Surface-to-volume ratio (**A**), compacity (**B**), entropy (**C**), correlation (**D**), coarseness (**E**), and complexity (**F**) showed significant differences (FDR = 0.022, 0.047, 0.045, 0.020, 0.034, and 0.034, respectively). PET, positron emission tomography; TMB, tumor mutational burden; FDR, false discovery rate.

**Figure 4:**
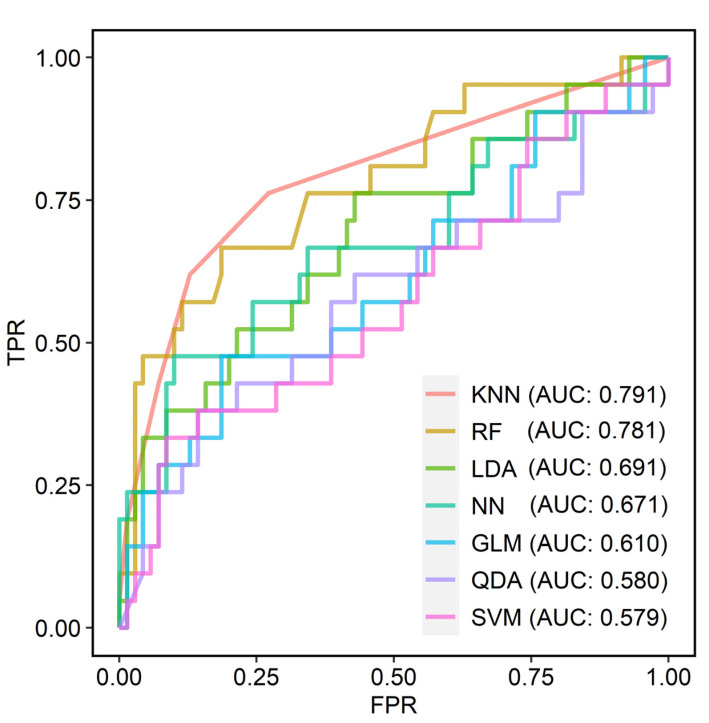
Discriminative performances of machine learning models for the prediction of TMB status by ROC curve analyses. The KNN model shows the best performance (AUC: 0.791), followed by RF (AUC: 0.781), LDA (AUC: 0.691), NN (AUC: 0.671), GLM (AUC: 0.610), QDA (AUC: 0.580), and SVM (AUC: 0.579) in ROC curve analysis. TMB, tumor mutational burden; ROC, receiver operating characteristic; AUC, area under curve; TPR, true positive rate; FPR, false positive rate; GLM, generalized linear model; LDA, linear discriminant analysis; QDA, quadratic discriminant analysis; KNN, k-nearest neighbors; SVM, support vector machine; NN, neural network; RF, random forest.

In correlation analysis, complexity (*r* = 0.332 and FDR = 0.007) and zone size non-uniformity (*r* = 0.351 and FDR = 0.011) showed significant correlations with TMB (Figure 2). Surface-to-volume ratio, compacity, entropy, correlation, coarseness, and complexity showed significant differences between the high- and low-TMB groups (Figure 3; FDR = 0.022, 0.047, 0.045, 0.020, 0.034, and 0.034, respectively). In univariable logistic regression analyses, surface-to-volume ratio, TLG, volume, area, compacity, complexity, entropy, correlation, coarseness, and zone size, non-uniformity showed significant discriminative power for the TMB group (Table 2). These 10 radiomic features were selected for the development of ML models. Among the seven ML algorithms, the RF model showed the highest accuracy (0.836), and the KNN model showed the largest AUC (0.791) in ROC curve analysis (Figure 4, Table 3).

### 3.3. Prognostic Validation of PET Radiomic Score

**Figure 5:**
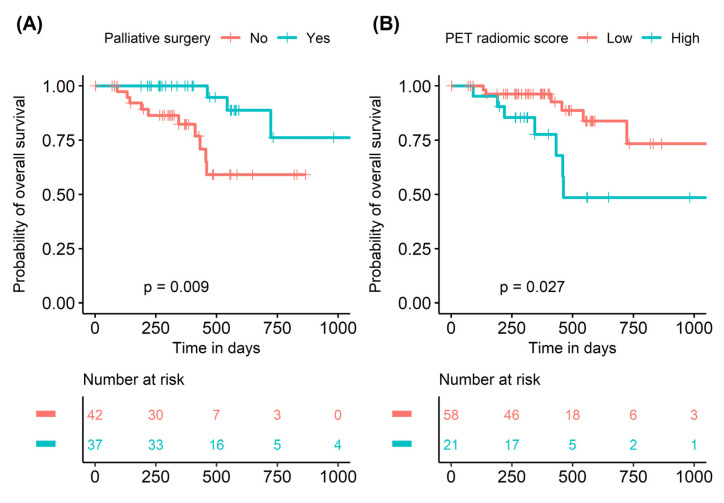
Kaplan–Meier survival curves of overall survival according to palliative surgery status (**A**) and PET radiomic score (**B**). PET, positron emission tomography.

In univariable Cox regression analysis for OS, palliative surgery status (HR = 0.199 and *p* = 0.015) and PET radiomic score (HR = 3.081 and *p* = 0.036) were significant prognostic factors (Table 4). TMB showed no prognostic significance for OS in either the continuous or the dichotomized variables. In multivariable Cox regression analysis for OS, both palliative surgery status (HR = 0.124, *p* = 0.008) and PET radiomic score (HR = 4.498, *p* = 0.046) were independent prognostic factors (Table 4). Kaplan–Meier survival curves showed significant survival differences according to palliative surgery status (2-year survival rate 58.1% vs. 76.1%, *p* = 0.007). Patients with a higher PET radiomic score showed significantly worse OS (2-year survival rate 48.6% vs. 73.3%; *p* = 0.027) than those with a lower PET radiomic score (Figure 5).

## 4. Discussion

In this study, several PET radiomics, including conventional PET parameters such as TLG and tumor volume as well as texture parameters such as entropy and coarseness, were revealed to have significant associations with TMB in CRC. Newly developed ML models were generally found to have good performance for the prediction of TMB. This study demonstrates that PET-based radiomics are useful image biomarkers for the prediction of TMB status. A PET radiomic score integrating significant imaging features has the potential to predict survival in patients with stage IV CRC.

CRC is a cancer subtype for which curative therapy for advanced or metastatic disease is actively performed. In patients with hepatic or pulmonary metastasis, resection of the metastatic tumor is considered to be a therapeutic option that is commonly followed by chemotherapy [2]. Palliative chemotherapy showed good survival benefits [4]. In addition, palliative surgery provides survival gain in non-curable colon cancer [25]. As 5-year survival of stage IV CRC patients is non-negligible (12–13 percent), it is valuable to predict survival in the palliative setting [26].

TMB is the total number of genetic mutations in a tumor tissue. As mutated genes can produce neo-antigens that are recognized by the immune cells, TMB is believed to be an excellent marker to predict tumor immunogenicity [27]. The present study demonstrated significant associations between several PET imaging features and TMB in CRC. Notably, entropy and zone size non-uniformity were higher in the high-TMB group than in the low-TMB group. These parameters reflect heterogeneity of the image: entropy indicates noisiness or randomness, and zone size non-uniformity indicates variance of homogeneous areas [28,29]. This result implies that high intratumoral heterogeneity is associated with high mutational burden. Two hypotheses are suggested, as follows. First, a large number of genetic mutations induces high variation in tumor characteristics, directly relating to high intratumor heterogeneity [30]. As many genetic mutations related to tumorigenesis have an effect on metabolism, high TMB can be related to metabolic heterogeneity. Second, tumor immunity is activated in tumors with high TMB via many neo-antigens [27]. Mixed cellular composition of various immune cells and tumor cells may cause metabolic heterogeneity in a tumor tissue. Furthermore, tumor volume and TLG were significant factors to predict high TMB. Although there have been previous studies to suggest a correlation between maximum SUV or other texture parameters and TMB [20,21], to the best of our knowledge, no previous study has demonstrated a relationship between volumetric parameters and TMB. This result is almost the same as a previous result suggesting that more genetic mutation corresponds with large tumor volume [31].

The developed ML models showed good discriminative performances for high TMB, suggesting that a combination of PET-based radiomics, not a single parameter, is helpful to predict TMB level. Measuring TMB requires an invasive procedure to obtain the tissue specimen and a high-cost genomic analysis. Therefore, TMB is not yet commonly acquired in clinical practice. The present study proposes the potential feasibility of PET-based radiomics to predict high TMB. Prediction of TMB status may be meaningful in itself because high TMB (≥10 Mut/Mb), not the absolute value of TMB, is the suggested criterion for immune therapy [32].

Although TMB showed no prognostic significance for OS in the present study, it is a prognostic biomarker for immunotherapy in various malignancies, including lung cancer and melanoma [33,34]. In several previous studies, the prognostic effect of TMB was revealed in CRC patients with conventional chemotherapy as well as immunotherapy [18,19]. However, the result of this study did not correspond with previous research. Several suppositions are proposed as follows. First of all, the subjects of this study were stage IV CRC patients, in contrast to the previous study, which enrolled CRC patients with surgery followed by adjuvant chemotherapy. In metastatic disease, various clinical factors such as metastatic extent or palliative surgery may affect the prognosis of patients. A good prognostic discrimination by palliative surgery status, which was demonstrated in this study, supported this hypothesis. Second, the treatment regimens were heterogeneous. Finally, a retrospective design with a small number of patients could be another reason or explanation for the differences in this study’s results.

The PET radiomic score as a surrogate image biomarker for TMB demonstrated prognostic value for OS. It has three significant implications. First, intratumoral heterogeneity was deemed to affect poor prognosis, considering that high PET radiomic score was related to features representing heterogeneity. It was well matched with a previous study that showed prognostic significance of the tumor heterogeneity parameter from FDG PET/CT in rectal cancer [35]. Second, high PET radiomic score reflected large tumor volume and metabolic tumor burden. Lee et al. reported that MTV and TLG were significantly independent prognostic factors in CRC [12]. Finally, the necessity of a simplified parameter to integrate various radiomic features was suggested. There were many varying features that could be extracted from the FDG PET/CT images. Furthermore, it was difficult to recognize the meaning of each texture parameter as mathematical products via complex calculation. An integrated single parameter, including anything from volumetric parameters to complicated texture parameters, was expected to be more useful in clinical applications. In this study, we developed the PET radiomic score by integrating various parameters based on association with TMB. It showed a good prognostic significance, suggesting the potential usefulness of an integrated single parameter from FDG PET/CT. Further study is warranted to evaluate clinical usefulness as a surrogate marker of TMB to assess eligibility for immunotherapy and predict immunotherapy response. To implement PET radiomic score in a clinical setting, initial FDG PET/CT and radiomic analysis should be performed to establish treatment plans for stage IV CRC patients. Also, clinicians may consider PET radiomic scores as supplementary information with other factors such as PD-L1 or TMB.

There were several limitations to this study. First, this retrospective study included patients who had both a pretreatment FDG PET/CT scan and information on TMB. It was surmised that many instances involved initiating treatment immediately based on only findings of pathology and CT or MRI before initial FDG PET/CT workup. Additionally, there were numerous referrals from external hospitals, leading to the assumption that there were many cases where an initial FDG PET/CT was not conducted. Therefore, the total number of patients included was relatively small, which may have introduced selection bias. Second, FDG PET/CT images from different scanners were employed. While we minimized batch effects through harmonization, future prospective studies with the same instrument may be necessary. Third, the performances of treatment options such as the chemotherapy regimen and palliative surgery were heterogeneous. Finally, there was no external validation. However, internal validation was conducted with five-fold cross validation to assess the generalizability of a model. A larger multicenter prospective study with the same treatment options would be warranted to validate our results.

In conclusion, a PET-based radiomic model was developed and validated for the prediction of TMB status in patients with stage IV CRC. The machine learning approach with KNN exhibited good performance in predicting the status of TMB. Various PET-based radiomics, including TLG, tumor volume, entropy, and coarseness, showed significant associations with TMB. The PET radiomic score, a single imaging biomarker integrating significant PET-based radiomics, has the potential to predict survival in stage IV CRC patients with palliative chemotherapy.

## Figures and Tables

**Table 1:** Clinical characteristics of patients.

Characteristics	Overall	Low TMB	High TMB	*p*
	(*n* = 91)	(*n* = 70)	(*n* = 21)	
Age (range, years)	58.0 (21–88)	56.8 (33–88)	62.1 (21–84)	0.154
Sex, male	53 (57.6%)	38 (54.3%)	15 (71.4%)	0.162
TMB	9.4 ± 12.1	5.7 ± 2.3	22.0 ± 2.3	0.002
Histological type				0.570
Adenocarcinoma	88 (96.7%)	68 (97.1%)	20 (95.2%)	
Small cell carcinoma	1 (1.1%)	1 (1.4%)	0 (0%)	
Unclassified carcinoma	2 (2.2%)	1 (1.4%)	1 (4.8%)	
Histological grade				0.430
Unknown	14 (15.2%)	9 (12.9%)	5 (23.8%)	
Well-differentiated	12 (13.0%)	11 (15.7%)	1 (4.8%)	
Moderately differentiated	53 (57.6)	41 (58.6%)	12 (57.1%)	
Poorly differentiated	12 (13.0%)	9 (12.9%)	3 (14.3%)	
Location				0.012
Colon	39 (42.9%)	25 (35.7%)	14 (66.7%)	
Rectum	52 (57.1%)	45 (64.3%)	7 (33.3%)	
Chemotherapy regimen				0.760
FOLFOX	58 (63.7%)	46 (65.7%)	12 (57.1%)	
FOLFIRI	25 (27.5%)	18 (25.7%)	6 (28.6%)	
Others	6 (6.6%)	5 (7.1%)	2 (9.5%)	
None	2 (2.2%)	1 (1.4%)	1 (4.8%)	
Palliative surgery				0.157
Yes	44 (48.4%)	31 (44.3%)	13 (61.9%)	
No	47 (51.6%)	39 (55.7%)	8 (38.1%)	

Note: Data are displayed as numbers of patients (proportion) or mean values ± standard deviation. Tumors with TMB greater than 10 were assigned to the high-TMB group. TMB, tumor mutational burden; FOLFOX, leucovorin, fluorouracil, and oxaliplatin; FOLFIRI, leucovorin, fluorouracil, and irinotecan.

**Table 2:** PET-based radiomics for the prediction of TMB status in univariable logistic regression analysis.

Features	Log (OR)	*p*	FDR
Surface-to-volume ratio	−13.33	0.006	0.036
Total lesion glycolysis	2.02 × 10^−6^	0.007	0.036
Volume	1.86 × 10^−5^	0.010	0.036
Area	9.93 × 10^−5^	0.012	0.036
Compacity	0.58	0.014	0.036
Complexity	8.48 × 10^−6^	0.004	0.019
Entropy	0.627	0.011	0.051
Correlation	6.20	0.006	0.051
Coarseness	−33.64	0.037	0.092
Zone size non-uniformity	0.003	0.002	0.037

FDR, false discovery rate; OR, odds ratio; TMB, tumor mutational burden.

**Table 3:** Performance of machine learning models for the prediction of TMB status.

Models	AUC	Accuracy	Sensitivity	Specificity	F1 Score	Precision
GLM	0.610	0.769	0.476	0.814	0.455	0.435
LDA	0.691	0.802	0.762	0.571	0.478	0.348
QDA	0.580	0.747	0.381	0.857	0.410	0.444
KNN	0.791	0.814	0.619	0.871	0.605	0.591
SVM	0.579	0.758	0.333	0.914	0.412	0.538
RF	0.781	0.836	0.667	0.814	0.583	0.519
NN	0.671	0.780	0.476	0.900	0.526	0.588

TMB, tumor mutational burden; AUC, area under curve; GLM, generalized linear model; LDA, linear discriminant analysis; QDA, quadratic discriminant analysis; KNN, k-nearest neighbors; SVM, support vector machine; NN, neural network; RF, random forest.

**Table 4:** Cox proportional hazards regression analysis of overall survival.

Variables	Univariable			Multivariable		
	HR	95% CI	*p*	HR	95% CI	*p*
Sex						
Female vs. Male	0.877	0.295–2.610	0.813	1.422	0.403–5.020	0.584
Age						
≥56	1.097	0.378–3.185	0.865	0.407	0.106–1.557	0.189
Location						
Colon vs. Rectum	1.023	0.352–2.974	0.966	1.050	0.282–3.904	0.942
Histological grade						
Well-differentiated						
Moderately differentiated	0.732	0.083–6.434	0.779	0		0.998
Poorly differentiated	1.947	0.212–17.886	0.556	5.389	0.424–68.475	0.194
Chemotherapy regimen						
FOLFOX						
FOLFIRI	0.324	0.067–1.561	0.160	0.315	0.056–1.778	0.191
Others	3.037	0.348–26.501	0.315	4.303	0.367–50.483	0.245
Palliative surgery						
Yes	0.210	0.057–0.772	0.019	0.123	0.026–0.594	0.009
TMB (continuous)	0.948	0.861–1.044	0.280			
TMB group						
Low vs. High	1.334	0.398–4.471	0.640	0.471	0.097–2.280	0.349
PET radiomic score						
Low vs. High	3.081	1.078–8.810	0.036	4.684	1.069–20.526	0.040

HR, hazard ratio; CI, confidence interval; TMB, tumor mutational burden; PET, positron emission tomography.

## Data Availability

The data used in this study are available from the corresponding author upon reasonable request.

## Supplementary Materials

The following supporting information can be downloaded at: https://www.mdpi.com/article/10.3390/cancers15153841/s1, Figure S1: Example of tumor segmentation with the Nestle adaptive threshold method; Table S1: Radiomic features included in the study.

## Correction Statement

The journal’s Editorial Office and Editorial Board are jointly issuing a resolution and removal of the Journal Notice linked to this article, as well as an update to the reviewer record. Following concerns raised about the integrity of the peer-review, the Editorial Office has conducted a post-publication peer-review of this article, supervised by an Editorial Board Member and involving an independent reviewer. As a result, Reviewer Report 2 was removed and replaced with two new reports. The review record has been updated accordingly. With this update, the Academic Editor is satisfied that the Editorial Process relating to this article has been completed as per MDPI’s Editorial Process policy. The Editorial Office would like to thank the authors for their collaboration during the process.
