# Supplementary material for: A Machine Learning Approach Using FDG PET-Based Radiomics for Prediction of Tumor Mutational Burden and Prognosis in Stage IV Colorectal Cancer
Source: Cancers (Basel). 2023 Jul 28;15(15):3841. doi: 10.3390/cancers15153841 (PMC10416826; doi:10.3390/cancers15153841)
Supplement: Supplementary file 1 [file cancers-15-03841-s001.zip › Figure S1.pdf]

SUPPLEMENTARY MATERIALS

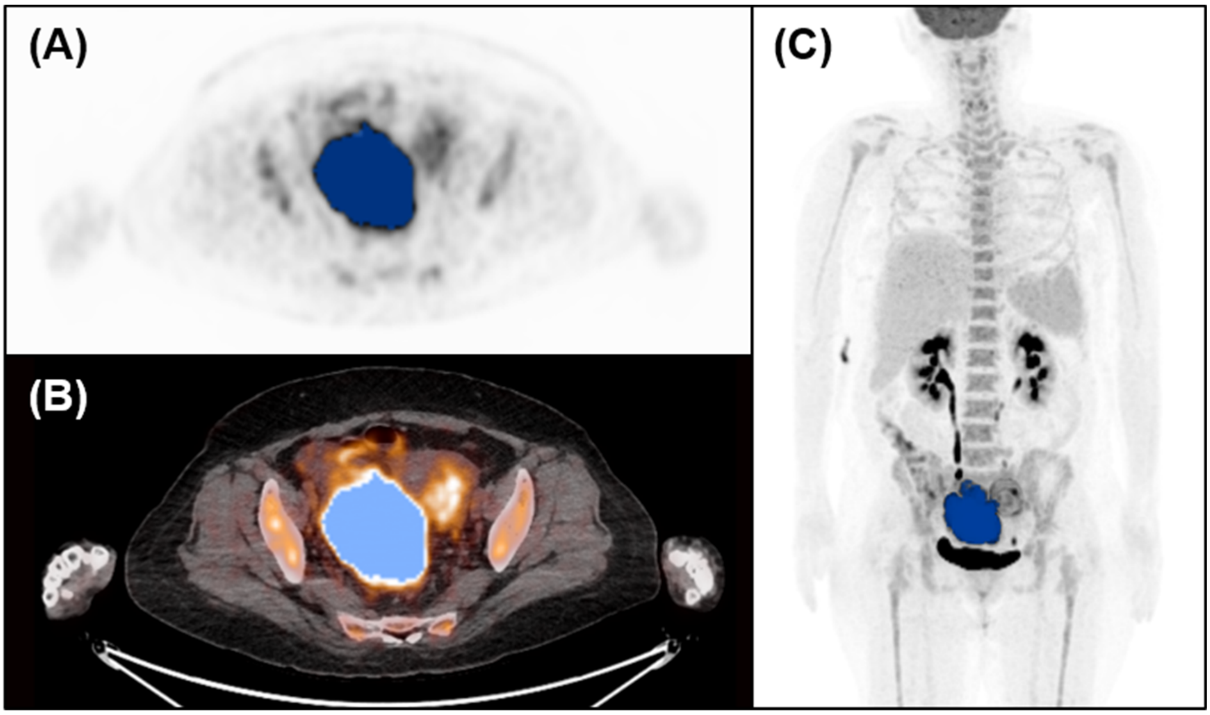

Supplementary Figure S1. Example of tumor segmentation with the Nestle adaptive threshold method

Volume of interest was delineated by the Nestle adaptive threshold method on PET images. Axial PET (A), axial fusion PET and CT (B), and maximum intensity projection images (C).
